# Acute Sleep Deprivation Impairs Motor Inhibition in Table Tennis Athletes: An ERP Study

**DOI:** 10.3390/brainsci12060746

**Published:** 2022-06-07

**Authors:** Lin Xu, Tao Song, Ziyi Peng, Cimin Dai, Letong Wang, Yongcong Shao, Lanxiang Wang, Xiechuan Weng, Mengfei Han

**Affiliations:** 1 School of Psychology, Beijing Sport University, Beijing 100084, China; rzxulin1997@126.com (L.X.); songtaozy@163.com (T.S.); pzyi121@163.com (Z.P.); 15071265226@163.com (C.D.); wlt2525@163.com (L.W.); 2 Rehabilitation Medicine Department of the 8th Medical Center of Chinese PLA General Hospital, Beijing 100091, China; 3 Department of Neuroscience, Beijing Institute of Basic Medical Sciences, Beijing 100850, China; 4 Aviation Psychology Research Office, Air Force Medical Center, Beijing 100142, China

**Keywords:** response inhibition, sleep deprivation, table tennis athletes, ERP, stop-signal task

## Abstract

Excellent response inhibition is the basis for outstanding competitive athletic performance, and sleep may be an important factor affecting athletes’ response inhibition. This study investigates the effect of sleep deprivation on athletes’ response inhibition, and its differentiating effect on non-athlete controls’ performance, with the aim of helping athletes effectively improve their response inhibition ability through sleep pattern manipulation. Behavioral and event-related potential (ERP) data were collected from 36 participants (16 table tennis athletes and 20 general college students) after 36 h of sleep deprivation using ERP techniques and a stop-signal task. Sleep deprivation’s different effects on response inhibition in the two groups were explored through repeated-measures ANOVA. Behavioral data showed that in a baseline state, stop-signal response time was significantly faster in table tennis athletes than in non-athlete controls, and appeared significantly longer after sleep deprivation in both groups. ERP results showed that at baseline state, N2, ERN, and P3 amplitudes were lower in table tennis athletes than in non-athlete controls, and corresponding significant decreases were observed in non-athlete controls after 36 h of sleep deprivation. Table tennis athletes showed a decrease in P3 amplitude and no significant difference in N2 and ERN amplitudes, after 36 h of sleep deprivation compared to the baseline state. Compared to non-athlete controls, table tennis athletes had better response inhibition, and the adverse effects of sleep deprivation on response inhibition occurred mainly in the later top-down motor inhibition process rather than in earlier automated conflict detection and monitoring.

## 1. Introduction

For a long time, the important role of sleep for athletes was overlooked [1]. In many cultures, less sleep represents greater self-discipline, and for athletes, limited sleep time is often repeatedly compressed by heavy training loads. At the same time, cross-time-zone competition, overly intense competition schedules, increased nighttime competitions, and mood swings such as excitement, tension, and anxiety prior to competition can all interfere with athletes’ circadian and homeostatic rhythms [2,3]. An increasing number of athletes report facing sleep deprivation and sleep disorders before and during competitions [4,5,6,7,8]. With technological advancement and the growth of medical research, it is gradually recognized that the prevalence of “sleep debt” can exhibit different degrees of adverse effects on athletes’ physiology, psychology, sports performance, and post-competition recovery [9,10,11,12,13,14,15,16,17]. More research is required to help us gain in-depth insight into the effects of lack of sleep on various aspects of athletes’ lives.

A key component of executive function is the ability to inhibit one’s responses in situations where the external environment or internal demands change and is important for dealing with complex external environments in a purposeful manner, in order to respond appropriately [18,19]. In table tennis, due to the confrontation across the net, the long holding time, the technical playing style, and other sports characteristics, the player must make fast and accurate observations and judgments of the incoming ball movement, direction, rotation, position, landing point, and other information in a limited time, and make the correct decision of whether to inhibit or change the customary action to return, which all rely on good reaction inhibition ability. Therefore, how to help athletes effectively improve response inhibition is an important issue that should be focused on in the field of competitive psychology.

The stop-signal task (SST) is the classical paradigm for research on inhibitory control. In SST, a stop signal usually occurs after several go signals. The time interval between the appearance of a go signal and the appearance of a stop signal is controlled by a predetermined probability. A complete SST consists of two relatively independent processes: the go process and stop process. In 1984, Logan et al. proposed a horse race model, which assumes that the success of an inhibition depends on the relative completion times of two independent processes: If the stop process is completed before the go process, then the participant can successfully inhibit the response after the stop signal appears; conversely, if the go process is completed before the stop process, then the participant cannot successfully inhibit the response after the stop signal appears [20]. In the SST, one of the most important dependent variables is stop-signal response time (SSRT), a criterion for assessing inhibitory control that represents the time it takes for an individual to “withdraw” a dominant response. It is often considered a relatively stable and automatic component of cognitive control, with shorter SSRT representing better inhibitory control [19,21].

Sleep is closely related to inhibitory control, and it is generally believed that sleep deprivation has adverse effects on response inhibition and flexibility [22,23,24]. For example, a functional magnetic resonance imaging (fMRI) study using the go/no-go paradigm found that 24 h of sleep deprivation caused a significant decrease in activation of the ventral and anterior prefrontal regions associated with inhibitory control [25]. Jin et al. measured the electroencephalogram (EEG) performance of 14 healthy men on response inhibition at baseline, after 12, 24, and 36 h of sleep deprivation, and after eight hours of recovery sleep, and found that sleep deprivation induced a dose-dependent functional decrease in response inhibition of prefrontal cortex activation by NoGo-N2 and NoGo-P3 [26]. According to the theory of brain plasticity, athletes who receive high-intensity special training for an extended period of time may have plastic changes in their cortical structure and functioning, compared to ordinary people [27,28]. Previous studies have documented the effects of sleep deprivation on response inhibition in the general population, but few studies have addressed the effects of sleep deprivation on response inhibition in this specific group of athletes. Our study addressed this gap by exploring the effect of sleep deprivation on response inhibition in athletes and how its differentiating effect on the performance of ordinary people is of great significance for improving athletes’ response inhibition ability through “sleep prescription”.

Through an extensive review of previous literature, we found that in the relevant research on the effects of sleep on athletes’ athletic performance and cognitive functioning, the above questions are mainly discussed by interview method, questionnaire method, and behavioral experiments, and event-related potential (ERP) technology has advantages which traditional behavioral experiments cannot match [7,10,17]. ERPs are part of the EEG signal which records the brain’s neural activity during wakefulness, sleep, and various task stimuli [29]. Due to its high temporal resolution, low price, and simple operation, ERP technology has become a powerful tool for measuring and evaluating neural activity in the brain. In studies on response control, ERP techniques have been shown to help elucidate the effects of stimulus variables on inhibitory control, with the most common EEG indicators being N2, P3, and error-related negativity (ERN) [30,31,32]. The N2 component of the frontal-central region is a negative component that usually peaks 200–350 ms after stimulus presentation and is the early stage of response inhibition, which can reflect the process and intensity of conflict monitoring and inhibition [33,34,35]. The P3 component of the central-top region, a positive component that appears at approximately 300–600 ms, is widely recognized as an important indicator of inhibitory control in stop-signal tasks (SST) and is closely related to the motion inhibition process [36]. Additionally, it can be localized to the pre-auxiliary motion region, which is a key part of the inhibitory control network [37,38,39]. The ERN is an anterior central negative component that peaks around 50–60 ms after the response, with the generating source located in the anterior cingulate cortex and the point of maximum wave amplitude at FCz [40,41]. ERN is associated with error detection, reflects the adjustment of short- and long-term response strategies after an error occurs, and is critical for adaptive and goal-directed action [35,42]. By analyzing these ERP indicators, we can better isolate the internal cognitive processes that respond to inhibiting external behavioral performance.

In laboratory experiments, many studies on sleep functionality in athletes have simulated chronic lack of sleep through total sleep deprivation [10]. This study uses ERP technology, combined with behavioral indicators, to explore more deeply the effects of sleep deprivation on response inhibition in table tennis athletes during an SST, by creating a 36-h complete sleep deprivation model. Three hypotheses are proposed in this study: (i) table tennis athletes have better inhibition abilities (shorter SSRT and higher stop accuracy) and use fewer mental resources (lower N2, ERN, and P3 amplitudes) than non-athlete controls in an SST; (ii) sleep deprivation can impair the response inhibition ability of table tennis players and non-athlete controls, as evidenced by slower response inhibition times, decreased correctness in behavioral indicators, and lower amplitudes of the relevant ERP components (N2, ERN, and P3) in the electrophysiological indices; (iii) due to table tennis athletes having received prolonged high-intensity specialized training, their response inhibition ability is less affected by sleep deprivation than non-athlete controls.

## 2. Materials and Methods

### 2.1. Participants

Thirty-six healthy male participants (age: 21.78 ± 2.19 years, including 16 table tennis athletes) from Beijing Sport University volunteered to participate in this experiment (See: Table 1). Participants were right-handed; had normal vision or corrected vision; were free of alcohol, caffeine, and drug addiction; and had good sleep quality as assessed by the Pittsburgh Sleep Quality Index. Conditions for inclusion in the control group were the following: (1) non-physical education college students; (2) no table tennis experience and no systematic professional table tennis training; (3) no regular exercise habits and less than 200 min of physical activity per week. Table tennis athletes were included considering the following conditions: (1) have received professional table tennis training; (2) were level II table tennis athletes in China; (3) maintained more than 10 h of table tennis training time per week. When the data analysis was performed, eight participants were excluded due to excessive head movement with too many EEG artifacts, the instrument malfunctioned during the experiment before completing the experimental procedure, or behavioral and ERP data were not collected simultaneously. The final sample consisted of 15 participants in the control group and 13 table tennis athletes, for a total of 28 participants (age: 21.79 ± 2.17 years) who were included in the data analysis. Each participant signed an informed consent form before participating in the experiment, the experiment was approved by the ethics committee of Beihang University, and participants were paid for the cost experiment at the end of the study period.

### 2.2. Experimental Design

**Figure 1:**
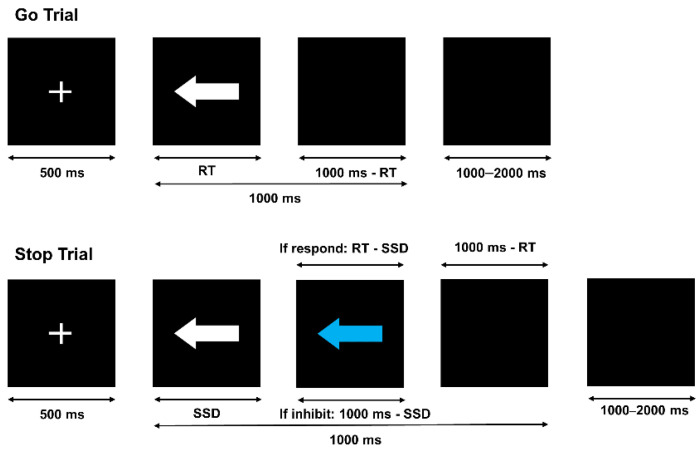
A schematic diagram of a stop-signal task (SST). The SST consisted of a go trial and stop trial. In the go trial, participants had to judge the direction of the white arrow (to the left or to the right) and press the corresponding button as accurately and quickly as possible (RT is the response time from the presentation of the go signal to making a keystroke response). In the stop trial, a stop signal (blue arrow) appeared after the go signal (the interval is indicated as stop-signal delay-SSD) and required the participant to stop responding.

This experiment was a 2 (group: Control vs. table tennis player) × 2 (sleep state: baseline state (BS) vs. sleep deprivation (SD)) mixed experimental design. Group was a between-subjects variable and sleep state was a within-subjects variable. An SST was used to investigate the effects of sleep deprivation on inhibitory control in athletes. The SST is the classical paradigm for studying response inhibition (schematic diagram of SST: Figure 1). The SST was divided into “go” trials and “stop” trials. In “go” trials, the go signal is a white left or right arrow, and the participant has to respond quickly and accurately with the corresponding keystroke according to the direction of the white arrow in the “go” trial. In the “stop” trial, the go signal appears first, and after the stop-signal delay (SSD, the time interval between the appearance of the go signal, and the appearance of the stop signal) a blue arrow (stop signal) appears which is identical to the white arrow except for its color. When the blue arrow to the left or right is seen, the participant needs to stop responding with the keystroke.

In the “go” trial, a 500 ms fixation point was first presented to remind the participants to focus their attention. The go signal (i.e., white arrow) was then presented in the left or right direction, and the participant needed to respond by pressing a key as soon as possible (“S” key for the left arrow, “K” key for the right arrow). The go signal disappeared after the key was pressed (followed by a 1000 ms-RT black screen) or disappeared automatically after 1000 ms. The randomly presented stimulus interval was 1000–2000 ms.

In the “stop” trial, a 500 ms fixation point was first presented to remind the participant to concentrate their attention. The stop signal (i.e., the blue arrow) appeared after the go signal, according to the SSD. According to the tracking procedure proposed by Logan et al., SSD changed dynamically with participants’ performance in the stop trial [43]. When a participant succeeded in inhibiting in the previous trial, the next trial SSD increased by 50 ms compared to the previous trial SSD, making response inhibition more difficult. Alternatively, when a participant failed to inhibit in the previous trial, the next trial SSD decreases by 50 ms compared to the previous trial SSD, making response inhibition easier. If the participant inhibited successfully, the stop signal was presented for 1000 ms-SSD; if the participant did not inhibit successfully, the stop signal was presented for RT-SSD, followed by a 1000-RT ms black screen. The randomly presented stimulus interval was 1000–2000 ms.

There were three blocks in the experiment, and each block had 42 go trials and 14 stop trials. The ratio of go trials to stop trials was 3:1, and there were 168 trials in total. The probability of the arrow going left or right was equal in the go trial and the stop trial.

### 2.3. Experimental Procedures

**Figure 2:**
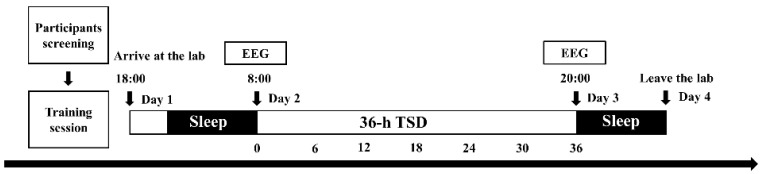
A flowchart of the four-day sleep deprivation experiment. Participants entered the laboratory at 18:00 on the first day of the experiment and slept in the laboratory that night under normal conditions. On the second day at 08:00, the first behavioral and EEG data acquisition of the stop-signal task was performed on the participants before the official start of the sleep deprivation experiment (SD: 0 h). On the third day at 20:00, the stop-signal task and sleep deprivation ended (SD: 36 h) with the acquisition of the second behavioral and EEG data. Participants remained in the laboratory for a recovery sleep on the evening of the third day and left the laboratory on the morning of the fourth day.

Participants who passed the screening were required to maintain adequate and regular sleep habits (go to bed before 11 pm, wake up before 8 am, and get at least eight hours of sleep each night) and keep a personal sleep diary for one week prior to the start of the experiment. The intake of alcohol, caffeine, and drugs was prohibited for one week prior to the experiment to prevent their interference with the sleep deprivation process. Participants arrived at the laboratory at 6 p.m. on the first day of the experiment, and experimenters informed participants about the procedure and the risks of sleep deprivation and explained the experimental tasks in detail. Participants practiced the experimental task after signing the consent form. Participants slept in the laboratory on the first night of the experiment. At 8 a.m. on the second day of the experiment, participants took baseline state measurements, and sleep deprivation officially began. During the experiment, participants sat in a shielded room with sound insulation and suitable lighting, and SST behavior and EEG data were recorded. During sleep deprivation, participants could engage in some non-strenuous recreational activities, such as reading, watching movies with calm plots, etc., and were prohibited from engaging in vigorous physical activities, as well as consuming alcohol and caffeinated beverages. Two participants entered the laboratory together for sleep deprivation in each experiment. The team supervised the participants in shifts throughout the sleep deprivation process to ensure that the participants remained awake, with two supervisors per shift. When the researchers found that the participants were sleepy and dozing off, they would keep the participants awake by talking to them, asking them to change their sitting position or allowing them to watch a movie for a while. At 8:00 p.m. on the third day of the experiment, the SST behavior and EEG data were recorded after 36 h of sleep deprivation, and the sleep deprivation ended. Participants underwent recovery sleep in the laboratory at night on the third day and left the laboratory on the morning of the fourth day (experimental flowchart is shown in Figure 2).

### 2.4. Data Acquisition and Analysis

#### 2.4.1. Behavioral Data Acquisition

We converted the E-Prime data file to Excel format to extract the behavioral indicators we were concerned about.

(1) Go RT: Time to correct response to the go trials.
(2) Go Accuracy: % correct responses in go trials.
(3) Mean SSD: The average SSD of all stop trials.
(4) Quantile RT (QRT): The response times of all the correct responses of the go trials were sorted in ascending order, and the go RT at the percentage of inhibition failure was taken as the QRT.
(5) SSRT: QRT-mean SSD.
(6) Stop Accuracy: % correct inhibited responses in stop trials.

In the behavioral data analysis, we focused on four behavioral indicators: Go RT, Go Accuracy, SSRT, and Stop Accuracy.

#### 2.4.2. ERP Data Acquisition

ERP data were acquired using a 32-channel amplifier and recorded online by Neuroscan software with a sampling rate of 1000 Hz. Electrodes were arranged in accordance with the extended international 10–20 system. FCz lies at the midpoint of the line connecting Fz and Cz. The data were analyzed offline using the EEGLAB and ERPLAB plug-ins, using average re-reference and band-pass filtering from 0.1 to 30 Hz after manually clipping the bad segments. The 50 Hz mains electricity was removed, and the eye-electricity artifacts were corrected using independent component analysis (ICA), and the artifact trials with amplitudes exceeding ±100 μV were removed from the artifact correction.

##### Analysis of N2 and P3 Amplitudes

The trials successfully inhibited in the stop trial were selected for inclusion in the ERP analysis and considering that the stop signal was preceded by the go signal, the mean values of 1100–900 ms before the presentation of the stop signal were selected for baseline correction. The average values of Fz, FCz, and Cz electrodes were selected to calculate the average amplitude of the N2 component (190–250 ms); the average values of F3, F4, Fz, FCz, and Cz electrodes were selected to calculate the average amplitude of the P3 component (250–400 ms).

##### Analysis of ERN Amplitude

Trials that failed to inhibit in the stop trial, and those that responded correctly in the go trial, were selected for inclusion in the ERP analysis. ERN was calculated by subtracting the response of the successful go signal from the response in the stop signal that failed to inhibit [44]. Using the moment of the response key press as the zero point, the average value of 250–50 ms before the key press was selected for baseline correction, and the peak amplitude of the FCz point was selected to calculate the ERN (0–150 ms) amplitude.

#### 2.4.3. Data Analysis

We compared the inhibitory control performance of table tennis athletes and non-athlete controls by analyzing SSRT, Stop Accuracy, go-RT, Go Accuracy, and ERP components (N2, ERN, and P3) amplitudes. The results of the Shapiro-Wilk (S-W) method showed that the data were approximately normally distributed. Because group was a between-subjects variable and sleep state was a within-subjects variable, a 2 (group: control vs. table tennis athletes) × 2 (sleep state: BS vs. SD) two-factor mixed-design ANOVA was performed on the behavioral indicators and average amplitudes of N2, P3, and ERN in the SST using IBM SPSS Statistics V22.0 (Armonk, NY, USA). Effect sizes for each ANOVA were estimated using partial eta squared (η^2^ *p*). Post hoc comparisons were conducted using the Fisher’s least significant difference (LSD) correction for multiple testing.

## 3. Results

### 3.1. Behavioral Performance

#### 3.1.1. SSRT

**Figure 3:**
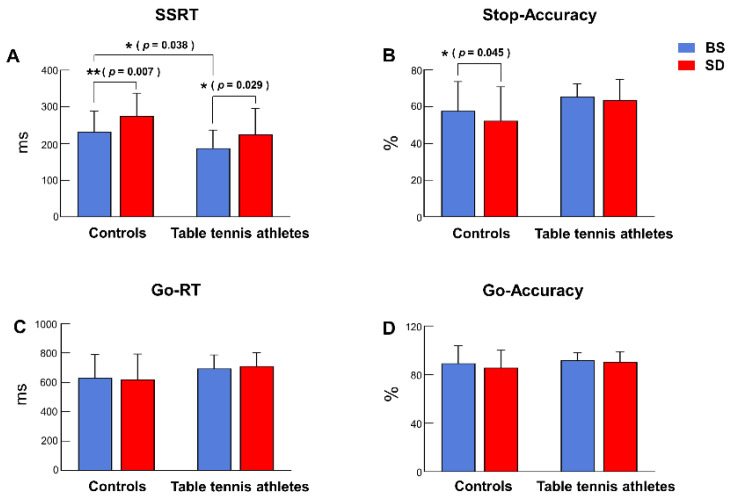
Response time and accuracy of behavioral indicators (mean ± SD). The blue bars represent the baseline state (BS), and the red bars represent the sleep deprivation (SD). (**A**) SSRT: Stop signal response time. (**B**) Stop Accuracy: % correct inhibited responses in stop trials. (**C**) Go RT: Time till correct response in the go trials. (**D**) Go Accuracy: % correct responses in go trials. * = *p* < 0.05, ** = *p* < 0.01.

Two-way ANOVA showed that the interaction between group and sleep state was not significant [F (1, 26) = 0.099, *p* = 0.756, η^2^ *p* = 0.004]. The main effect of sleep state was significant [F (1, 26) = 13.665, *p* = 0.001, η^2^ *p* = 0.345], and SSRT was longer after sleep deprivation compared to the baseline state. The main effect of group was significant [F (1, 26) = 5.687, *p* = 0.025, η^2^ *p* = 0.179], and table tennis athletes have shorter SSRT. The results of post hoc tests showed that the SSRT of table tennis athletes were shorter than that of the control group in the BS condition (*p* = 0.038). After SD, significant prolongation of SSRT was observed in both table tennis athletes (*p* = 0.029) and controls (*p* = 0.007) (Figure 3, Table 2).

#### 3.1.2. Stop Accuracy

The interaction between group and sleep state was not significant [F (1, 26) = 0.844, *p* = 0.367, η^2^ *p* = 0.031]. The main effect of sleep state was marginally significant [F (1, 26) = 3.828, *p* = 0.061, η^2^ *p* = 0.128], and stop-accuracy decreased after sleep deprivation. Additionally, the main effect of the group was not significant [F (1, 26) = 3.498, *p* = 0.073, η^2^ *p* = 0.119]. Post hoc tests revealed that in the BS condition, the differences between the table tennis athletes and the control group were not significant (*p* = 0.118). After SD, stop-accuracy showed a significant decrease in the control group (*p* = 0.045), and table tennis athletes did not differ significantly (*p* = 0.485) (Figure 3, Table 2).

#### 3.1.3. Go-RT

For the go-RT, the interaction between group and sleep state was not significant [F (1, 26) = 0.771, *p* = 0.388, η^2^ *p* = 0.029]. The main effects of sleep state [F (1, 26) = 0.064, *p* = 0.802, η^2^ *p* = 0.002] and group [F (1, 26) = 2.463, *p* = 0.129, η^2^ *p* = 0.087] were not significant. The post hoc tests showed no significant differences between the table tennis athletes and the control group in the BS condition (*p* = 0.207). There were no significant differences in go-RT before and after sleep deprivation between the table tennis athletes (*p* = 0.447) and the control group (*p* = 0.651) (Figure 3, Table 2).

#### 3.1.4. Go Accuracy

Two-way ANOVA showed that the interaction between group and sleep state was not significant [F (1, 26) = 0.502, *p* = 0.485, η^2^ *p* = 0.019]. The main effects of sleep state [F (1, 26) = 1.773, *p* = 0.195, η^2^ *p* = 0.064] and group [F (1, 26) = 0.747, *p* = 0.395, η^2^ *p* = 0.028] were not significant. The results of the post hoc tests showed no significant differences between the table tennis athletes and the control group in the BS state (*p* = 0.606). After sleep deprivation, there was no significant difference in go-accuracy between the table tennis athletes (*p* = 0.674) and the control group (*p* = 0.146) (Figure 3, Table 2).

### 3.2. ERP Component Amplitude

#### 3.2.1. N2 Component

**Figure 4:**
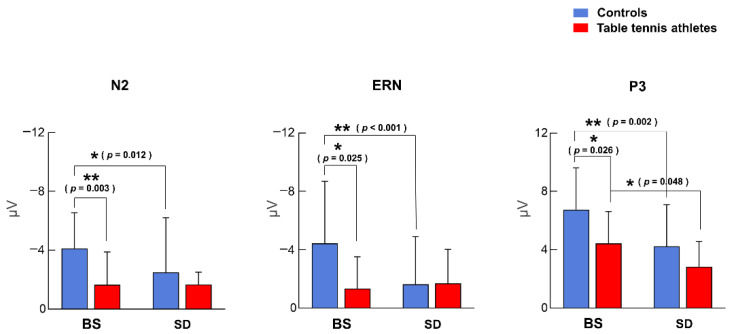
N2, ERN, and P3 component average amplitudes (mean ± SD). The blue bars represent the control group, and the red the table tennis athletes. * = *p* < 0.05, ** = *p* < 0.01.

**Figure 5:**
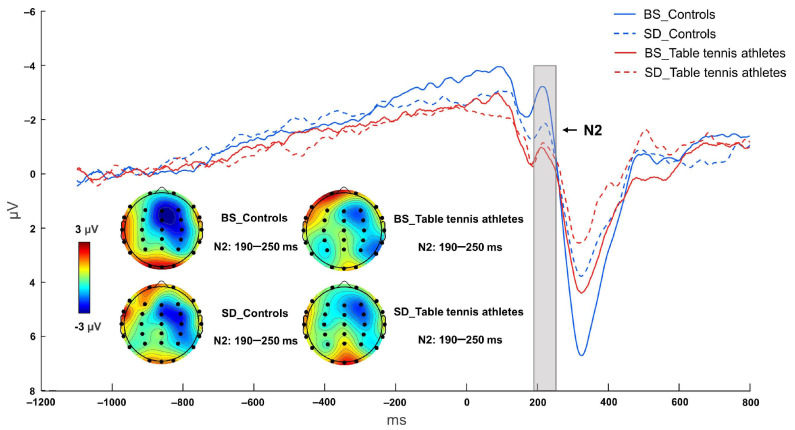
Grand mean ERP amplitude of table tennis athletes and controls on N2 in baseline state (BS) and 36 h sleep deprivation (SD) conditions. The data were averaged from Fz, FCz, and Cz. The topographies correspond to average activity in the time windows (190–250 ms, indicated by the gray bar) around the local peaks.

Two-way ANOVA showed that the interaction of sleep state and group was not significant [F (1, 26) = 3.455, *p* = 0.074, η^2^ *p* = 0.117]. The main effect of the group was significant [F (1, 26) = 4.833, *p* = 0.037, η^2^ *p* = 0.157], and the control group had a larger N2 negativity peak than the table tennis athletes. The main effect of sleep state [F (1, 26) = 3.306, *p* = 0.081, η^2^ *p* = 0.113] was not significant. In the BS condition, the control group had a larger peak of negativity than the table tennis athletes (*p* = 0.003). The control group in the BS condition also had a larger negativity peak compared to the SD condition (*p* = 0.012). However, N2 amplitude had no significant (*p* = 0.978) difference before and after sleep deprivation in table tennis athletes (Figure 4 and Figure 5, Table 3).

In the framework of traditional Null Hypothesis Statistical Testing (NHST), the hypothesis test is performed only under the assumption that H0 is true and cannot provide evidence for why H0 is true. The Bayes factor represents the ratio between the strength of support for the null hypothesis and the alternative hypothesis in the current data, which can more adequately support the null hypothesis compared to NHST [45]. Since NHST did not detect significant changes in N2 amplitude in the table tennis athletes before and after sleep deprivation, to detect whether there was really no difference in N2 amplitude in table tennis athletes before and after sleep deprivation, we supplemented the NHST with a Bayes factor for the test of difference in N2 amplitude in table tennis athletes.

Using JASP [46] to calculate Bayesian factors with default prior width, the data were more likely under the alternative model compared to the null model. We interpreted Bayes factors (BF_01_) of <3 as anecdotal, 3–10 as substantial, 10–30 as strong, 30–100 as very strong, and >100 as decisive evidence [47]. Bayesian statistics showed that BF_01_ = 3.592 for the difference in N2 amplitude in table tennis athletes before and after sleep deprivation, indicating that the current data were 3.592 times more likely to occur under the null hypothesis than under the alternative hypothesis. According to the classification criteria proposed by Jeffreys [47], there is moderately strong evidence to support the null hypothesis, which states that there is no difference in N2 amplitude in table tennis athletes before and after sleep deprivation.

#### 3.2.2. ERN Component

**Figure 6:**
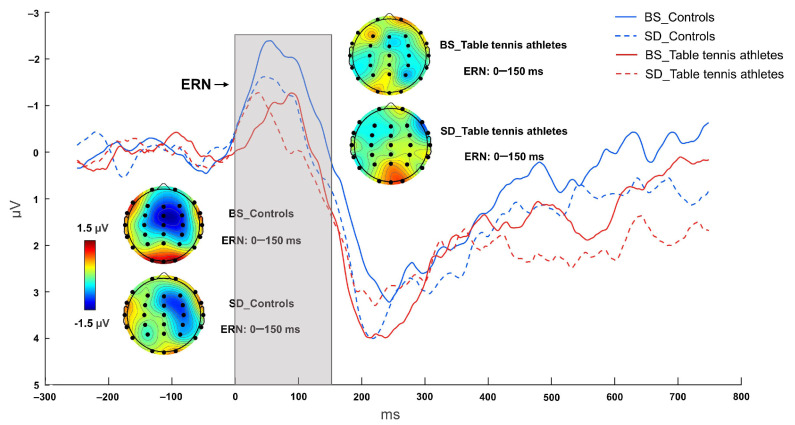
Grand mean ERP amplitude of table tennis athletes and controls on ERN in baseline state (BS) and 36 h sleep deprivation (SD) conditions. The data from FCz. The topographies correspond to average activity in the time windows (0–150 ms, indicated by the gray bar) around the local peaks.

The interaction between sleep state and group was significant [F (1, 26) = 11.887, *p* = 0.002, η^2^ *p* = 0.314]. The main effect of sleep state was significant [F (1, 26) = 7.054, *p* = 0.013, η^2^ *p* = 0.213], and the BS condition had a larger negative ERN peak than the SD condition. The main effect of group was not significant [F (1, 26) = 1.888, *p* = 0.181, η^2^ *p* = 0.068]. The results of post hoc tests showed that in the BS condition, the control group had larger ERN amplitudes than the table tennis athletes (*p* = 0.025). The control group had a larger negative ERN peak in the BS condition compared to the SD condition (*p* < 0.001) (Figure 4 and Figure 6, Table 3). However, for table tennis athletes, there was no significant difference in ERN amplitude between the BS and SD conditions (*p* = 0.593, BF_01_ = 3.236); the current data were 3.236 times more likely to occur under the null hypothesis than under the alternative hypothesis; and there was moderately strong evidence to support there being no difference in ERN amplitude in table tennis athletes before and after sleep deprivation.

#### 3.2.3. P3 Component

**Figure 7:**
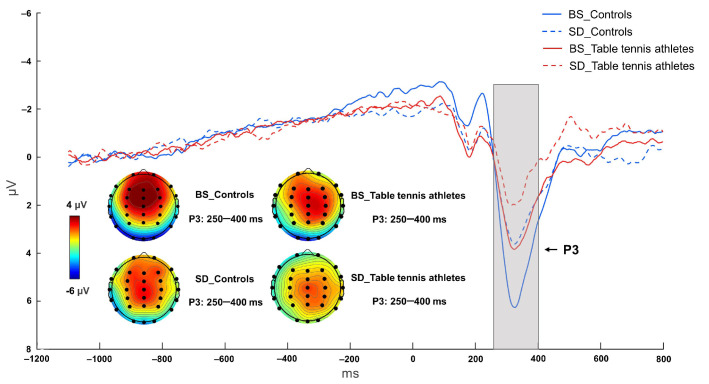
Grand mean ERP waveforms of table tennis athletes and controls on P3 in baseline state (BS) and 36 h sleep deprivation (SD) conditions. The data were averaged from F3, F4, Fz, FCz, and Cz. The topographies correspond to average activity in the time windows (250–400 ms, indicated by the gray bar) around the local peaks.

The interaction between group and sleep state was not significant [F (1, 26) = 0.689, *p* = 0.414, η^2^ *p* = 0.026]. The main effect of sleep state was significant [F (1, 26) = 14.970, *p* = 0.001, η^2^ *p* = 0.365], and the BS condition had a larger P3 peak than the SD condition. The main effect of group was also significant [F (1, 26) = 5.688, *p* = 0.025, η^2^ *p* = 0.179], the control group had a larger P3 amplitude than the table tennis athletes. Post hoc tests showed that the control group in the BS condition had a significantly larger P3 peak than the table tennis athletes (*p* = 0.026). Table tennis athletes (*p* = 0.048) and controls (*p* = 0.002) both showed a significant decrease in P3 amplitude after sleep deprivation (Figure 4 and Figure 7, Table 3).

### 3.3. Correlation between EEG and Behavioral Data

#### 3.3.1. N2 Amplitude and SSRT

**Figure 8:**
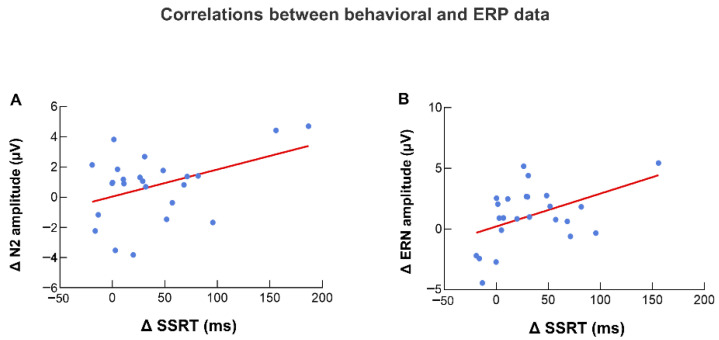
Correlations between behavioral and ERP data. (**A**) Correlation between ΔN2 amplitude (SD-BS) and ΔSSRT (SD-BS). (**B**) Correlation between ΔERN amplitude (SD-BS) and ΔSSRT (SD-BS).

Data from both groups of participants were combined and a correlation analysis was performed on ΔSSRT (SD-BS) and ΔN2 amplitude (SD-BS) after removing four extreme values to check for consistency between behavioral changes and ERP changes after sleep deprivation. The correlation analysis showed a significant positive correlation between ΔSSRT and ΔN2 (r = 0.421, *p* = 0.040, Figure 8).

#### 3.3.2. ERN Amplitude and SSRT

Data from both groups of participants were combined and a correlation analysis was performed on ΔSSRT (SD-BS) and ΔERN amplitude (SD-BS) after removing four extreme values. The correlation analysis showed a significant positive correlation between ΔSSRT and ΔERN (r = 0.459, *p* = 0.024, Figure 8).

#### 3.3.3. P3 Amplitude and SSRT

Data from both groups of participants were combined and a correlation analysis was performed on ΔSSRT (SD-BS) and ΔP3 amplitude (SD-BS) after removing three extreme values. The correlation analysis showed that the correlation between ΔSSRT and ΔP3 was not significant. (r = −0.030, *p* = 0.887).

## 4. Discussion

In this study, we used ERP technology and combined behavioral indicators to investigate the effects of sleep deprivation on response inhibition in table tennis athletes and non-athlete controls. The findings revealed that table tennis athletes showed better response inhibition than non-athlete controls in the SST, and in the baseline state, table tennis athletes had shorter SSRT and lower N2, P3, and ERN amplitudes than the control group. After sleep deprivation, SSRT was significantly prolonged in both table tennis athletes and non-athlete controls, demonstrating the impairing effect of sleep deprivation on response inhibition. Results consistent with behavioral indicators were also reflected in ERP indicators, with significant decreases in P3 amplitude after 36 h of sleep deprivation in both table tennis athletes and non-athlete controls. However, the two groups of participants showed dissociated results in N2 and ERN amplitudes; N2 and ERN amplitudes showed significant decreases after sleep deprivation in non-athlete controls but did not differ significantly before and after sleep deprivation in table tennis athletes, suggesting that the adverse effects of sleep deprivation on response inhibition in table tennis athletes may mainly impair top-down control of motor inhibition.

According to the assumptions of the horse-race model [20,48,49], poor inhibitory control may be due to too fast a response to the go signal or too slow a response to the stop signal. Too fast a response to the go signal can cause the fast response to the go signal to be executed before the response to the stop signal is made, resulting in a failure of inhibition. Although go-RT did not reach statistically significant differences by group, table tennis athletes exhibited longer go-RT both before and after sleep deprivation compared to the general athletes. This may indicate that the table tennis athletes did not tend to react quickly in the execution of control, and consequently traded off speed for accuracy. This may be related to the sport characteristics of table tennis with its small ball, racket, and tabletop. If you pursue speed, you may not be able to ensure that the ball is hit on the table, and once it is out of bounds, it will result in losing points or even the whole game. This requires table tennis athletes to not only have speed, but also to ensure the drop of the receiving serve, a sport characteristic that has affected table tennis athletes in the execution control process during long-term training and competition. After sleep deprivation, neither non-athlete controls nor table tennis athletes found significant changes from baseline status on go- accuracy and go-RT, which may be due to the fact that the go process task is less difficult and not sensitive to the effects caused by sleep deprivation.

During the stop process, the impairment of 36 h of sleep deprivation on the response inhibition ability of table tennis athletes and non-athlete controls by SSRT, a representative indicator of response inhibition, could clearly be observed. After sleep deprivation, SSRT was significantly prolonged and inhibitory processing was slowed in both groups of participants, and when the go and stop processes competed, there was no guarantee that the stop process could be completed before the go process, resulting in a failure of inhibition. The performance of the stop accuracy was also consistent with the SSRT, with a significant decrease in stop-trial accuracy occurring in the general college participants after sleep deprivation. Although table tennis athletes did not reach a statistically significant level, they also exhibited a trend of decreased accuracy after sleep deprivation. These results demonstrate that sleep deprivation leads to slower inhibition processing and reduced inhibition accuracy. In the baseline state, the SSRT of table tennis athletes was significantly shorter than that of the average college student, which might prove that table tennis athletes have a faster inhibitory processing speed than non-athlete controls in the SST. This is also consistent with previous results, where Wang et al. found shorter SSRTs in tennis athletes compared to a sedentary control group in an SST, and which also found better response inhibition in open skill sports (e.g., table tennis, badminton, tennis, etc.) than in closed skill sports (e.g., running, swimming, etc.) [50]. Similar results have been observed in other sports such as soccer, baseball, and basketball [51,52,53], all of which support the idea that long-term athletic training improves athletes’ inhibitory control.

This study’s most important finding was that sleep deprivation has different effects on the response inhibition abilities of table tennis athletes and non-athlete controls. Sleep deprivation reduced the amplitudes of N2 and ERN in the SST in non-athlete controls, whereas no differences in N2 and ERN were observed in table tennis athletes before and after sleep deprivation. Combining the performances of table tennis athletes and non-athlete controls at baseline, the results suggest that this difference may be related to table tennis athletes’ advanced “neural efficiency” due to prolonged training [54,55]. The neural efficiency hypothesis states that individuals with excellent performance display lower (more efficient) brain activation while performing cognitive tasks [56]. A recent meta-analysis pointed to neural efficiency as one of the prominent neural processing characteristics found in athletes, with athletes having better behavioral performance compared to novices or non-athletes while recruiting fewer neural resources to perform motor and cognitive tasks [57]. The advantage of athletes over non-athletes suggests that motor experience has a positive effect on neurological functioning and behavioral performance.

In tasks related to response inhibition, N2 and ERN are often considered as ERP components associated with automatic processing [21]. Research has suggested that the N2 component in the inhibitory control task reflects the monitoring of conflicting information and the allocation of attentional resources to better cope with the ensuing response inhibition [33,58,59]. The ERN, like the N2 component, is a negative ERP component which appears early in inhibitory control and reflects the automated perceptual processes of early response errors [60,61]. The amplitude of the N2 component of the prefrontal-central region and the ERN component from the anterior cingulate cortex were lower in table tennis athletes than in controls at the baseline state, indicating a tendency to conserve neural resources devoted to conflict monitoring and error processing during pre-response inhibition in table tennis athletes [30]. This may be related to the fact that athletes have been engaged in special training for a prolonged period of time, and in sports situations such as table tennis and other fast-response sports, there is a high demand for conflict monitoring, and only timely and accurate detection of conflict can enable them to adjust their reactions in time according to the characteristics of the opponent’s stroke. While non-athlete controls need to invest a lot of attention resources in order to be able to accurately identify and respond to conflicts in a timely manner, the monitoring of conflicts by table tennis athletes has shifted from the initial need to invest a lot of attention resources to a more bottom-up processing with a higher degree of automatic processing and a gradually decreasing need for central attention resources.

An fMRI study that recruited 14 table tennis athletes and 14 general students also confirmed that table tennis athletes showed lower activation of cortical areas in early sensory information processing, information matching recognition, and response selection compared to the general population [62]. A recent review study also noted that athletes performed more successfully than novices when faced with unexpected situations that required reactions to impending events, recruiting fewer attention resources, and devoting more attention to subsequent goal analysis [63].

In a study by Kusztor et al. [21], it was noted that cognitive processing in early bottom-up automation appears to be less affected by sleep deprivation compared to more advanced top-down cognitive control. The process of sleep deprivation is accompanied by significant changes in fatigue status [64,65,66]. Furthermore, 36 h of sleep deprivation caused participants to experience mental fatigue and reduced central nervous system arousal, leading to impaired sustained attention [67]. For the general college student population, impaired attention cannot be sustained by automated, bottom-up control alone to detect and monitor conflict, requiring constant investment of additional cognitive resources to sustain response inhibition. The higher level of automated processing that table tennis athletes have can help them devote more cognitive resources to more advanced cognitive processing.

P3 is considered a more reliable marker of response inhibition than N2 and is strongly associated with motor inhibition [30,31,36,68,69]. It also reflects, to some extent, the functions of information integration, attentional regulation, resource allocation, and post-response adaptation, representing more advanced top-down cognitive control processes [21]. Consistent with the results of N2 and ERN, the lower P3 amplitude evoked by table tennis athletes in the baseline state compared to general college participants reflects the fact that long-term table tennis training significantly improves the neural efficiency of inhibitory processing, thereby compressing the associated cognitive resources and minimizing the control resources that athletes spend on the inhibitory task. After sleep deprivation, both table tennis athletes and non-athlete controls showed a significant decrease in the amplitude of the P3 component, indicating that 36 h of sleep deprivation produced a top-down disintegration of the late process of response inhibition. It is possible that sleep deprivation adversely affects the response control of table tennis athletes by impairing the motor inhibition process represented by the P3 component. This is also consistent with previous findings, as Qi et al. found that the early N1 component amplitude and latency did not change after 43 h of sustained wakefulness, while the P3 component showed a decrease in amplitude and prolonged latency in the no-go condition [70]. Liu et al. performed 72 h of sleep deprivation on 12 healthy male participants and found that no significant changes in N2 amplitude were observed during the execution of the go/no-go task, while P3 amplitude exhibited a significant decrease [71]. These results are evidence that later, more advanced top-down cognitive control is more sensitive to sleep deprivation compared to early automated control.

Regular sleep is a prerequisite for human survival and plays a vital role in ensuring physical and mental health [72]. For athletes, sleep is an important part of training and pre-competition preparation, and good sleep is not only fundamental for athletes to maintain optimal competitive performance, but also important for reducing the risk of injury and illness [8,17,73]. Our findings show that when athletes train and compete with a “sleep debt,” even though they can notice incoming stimuli and detect unexpected situations or changes in the opponent’s movements on the field, they still have difficulty suppressing inappropriate dominant responses and cannot necessarily respond correctly to such unexpected situations in a timely manner.

Good sleep shapes better athletes, and it is generally recommended that athletes should get between seven and nine hours of sleep to ensure adequate physical and mental recovery after training [74]. Meanwhile, during training and competitions, athletes can get better quality sleep by maintaining a dark sleep environment [75], avoiding caffeine intake before bedtime [76], going to sleep, and waking up at regular times [77], and avoiding electronic screens before bedtime [78], among others.

Although our study provides ERP evidence that sleep deprivation impairs response inhibition, we must acknowledge that the present study still has some limitations. (1) In terms of the study population, the study’s participants were limited to young male table tennis level II athletes in China. Consequently, the following questions arise: Does sleep deprivation have different effects on elite athletes? Are our findings generalizable across sex and age? These questions still need to be followed up with more in-depth exploration. (2) In terms of methodological tools, the high temporal resolution of EEG signals can directly reveal the temporal characteristics of cognitive processing in the neural cortex of the brain; however, the limitations of spatial resolution cannot precisely provide the characteristics of neuronal or cluster activities with high spatial resolution. In future studies, a combination of techniques such as EEG and ERPs with imaging techniques such as positron emission tomography and fMRI with high spatial resolution can be considered to further reveal the effects of sleep deprivation on response inhibition in athletes. (3) In terms of circadian rhythms, due to various practical conditions, we performed baseline tests on EEG and behavior at 8:00 before sleep deprivation and post-tests at 20:00 at the end of sleep deprivation period. Circadian rhythms may influence cognitive performance after sleep deprivation. The decline in cognitive performance due to sleep deprivation is usually most severe at the nadir of the circadian rhythm (approximately 6:00 to 8:00) and tends to recover partially around 16:00 to 20:00 as the circadian rhythm rises [79]. In future studies, tighter control to match circadian rhythms at different test times is required.

## 5. Conclusions

The present study used an SST to provide ERP evidence that 36 h of sleep deprivation impairs response inhibition in table tennis athletes. Sleep deprivation had different effects on response inhibition in table tennis athletes and non-athlete controls, with sleep deprivation decreasing the amplitude of the P3 component in both groups of participants but segregating the N2 and ERN components. The amplitudes of N2 and ERN in non-athlete controls showed significant decreases after sleep deprivation, whereas table tennis athletes did not show significant differences in N2 and ERN components after sleep deprivation. This may be due to the tendency of table tennis athletes to conserve neural resources devoted to conflict monitoring and error processing during the pre-response inhibition process, with a higher level of automated processing compared to the average college student. Sleep deprivation impaired higher top-down cognitive processing in both groups of participants, suggesting that later higher top-down cognitive control is more sensitive to sleep deprivation than early automatic control.

## Figures and Tables

**Table 1:** Demographic information of each group (M ± SD).

Group	Controls	Athletes	*p*-Value
Number	20	16	
Gender	Male	Male	
Age	21.25 ± 2.15	22.44 ± 2.13	0.107
Education	14.90 ± 1.77	15.88 ± 1.89	0.121
Habitual sleep time	7.27 ± 0.74	7.03 ± 1.02	0.421
Physical activity	<200 min of physical activity per week	>10 h training time per week	

**Table 2:** Means and standard deviations of the behavioral data listed for all conditions (BS_Controls, BS_Table tennis athletes, SD_Controls, SD_Table tennis athletes).

	BS		SD	
	Controls	Athletes	Controls	Athletes
SSRT (ms)	231.806 ± 56.433	187.717 ± 49.138	275.732 ± 61.761	224.755 ± 70.910
Stop-ACC (%)	57.672 ± 16.027	65.452 ± 7.073	52.349 ± 18.465	63.531 ± 11.468
Go-RT (ms)	628.795 ± 160.636	694.308 ± 92.807	619.496 ± 173.676	711.154 ± 91.855
Go-ACC (%)	89.387 ± 14.360	91.639 ± 6.329	85.797 ± 14.247	90.543 ± 8.399

**Table 3:** Means and standard deviations of ERP amplitudes’ means listed for all conditions (BS_Controls, BS_Table tennis athletes, SD_Controls, SD_Table tennis athletes).

	BS		SD	
	Controls	Athletes	Controls	Athletes
N2 (μV)	−4.353 ± 2.119	−1.648 ± 2.235	−2.481 ± 3.729	−1.668 ± 0.834
ERN (μV)	−4.447 ± 4.238	−1.332 ± 2.184	−1.634 ± 3.262	−1.698 ± 2.328
P3 (μV)	6.745 ± 2.868	4.448 ± 2.164	4.231 ± 2.859	2.822 ± 1.758

## Data Availability

The datasets generated for this study are available on request to corresponding authors.
